# Nutritional Profile of Commercialized Plant-Based Meat: An Integrative Review with a Systematic Approach

**DOI:** 10.3390/foods12030448

**Published:** 2023-01-18

**Authors:** Bernardo Romão, Raquel Braz Assunção Botelho, Maria Luiza Torres, Dayanne da Costa Maynard, Maria Eduarda Machado de Holanda, Vinícius Ruela Pereira Borges, António Raposo, Renata Puppin Zandonadi

**Affiliations:** 1 Department of Nutrition, University of Brasília, Brasília 70910-900, Brazil; 2 Faculty of Health Sciences, University Center of Brasilia (CEUB), Brasília 70790-075, Brazil; 3 Department of Computer Sciences, University of Brasília, Brasília 70910-900, Brazil; 4 CBIOS (Research Center for Biosciences and Health Technologies), Universidade Lusófona de Humanidades e Tecnologias, Campo Grande 376, 1749-024 Lisboa, Portugal

**Keywords:** plant-based, meat substitutes, nutritional composition, ingredients

## Abstract

Given the high cost of production of animal-based meats and the increase in the number of adepts of meatless diets, the need for plant-based meat substitutes is growing. In this prosperously growing market, there is a lack of knowledge about the nutritional value of these meat substitutes and their ingredients. This study aims to review the nutritional composition and ingredients of meat substitutes commercialized worldwide. An integrative review was performed with a systematic literature search in PubMed, EMBASE, Scopus, Science Direct, Web of Science, and 11 studies were selected to compose the sample of this review. Data on meat substitutes’ nutritional composition and ingredients from different categories were collected and analyzed. The results showed that meat substitutes commonly present lower energy values and higher amounts of carbohydrates and dietary fiber. Protein values varied according to the meat substitute category, with some showing a higher concentration than others, more specifically in substitutes for bovine meat. Higher values were found in the Pieces category and lower in Seafood substitutes. Unlike animal meat, vegan meat has a proportion of carbohydrates higher than protein in most samples, except for chicken substitutes. Meat substitutes presented similar total and saturated fat content compared to their animal-based counterparts. Higher amounts of fat were found in the “Various” category and lower in “Pieces”. Ingredients such as soy, pea, and wheat were the primary protein sources in meat substitutes, and vegetable oils were their primary fat source. Methylcellulose, various gums, and flavorings were the most used food additives. In general, meat substitutes presented high concentrations of sodium, possibly collaborating with an excessive sodium intake, highlighting the need for developing sodium-reduced or sodium-free alternatives. Most of the included samples did not describe the concentration of iron, zinc, and vitamin B12. Further studies are needed to develop meat substitutes with better nutritional composition, fulfilling the need for equivalent substitutes for animal-based meat.

## 1. Introduction

The demand for plant-based meat substitutes is growing worldwide for several reasons, such as welfare, sustainability, and health benefits [1,2]. Meat is a food that is ostensibly present in the eating habits of western populations, being responsible for providing several key nutrients such as proteins, fats, minerals such as iron and zinc, and vitamins A and B12 [3]. Its world consumption is about 25 kg per capita per year [3]; however, its production can harm the local environment and world sustainability.

Meat production demands the concomitant use of a series of resources, such as land, water, and energy, and this model has already proven to be economically unfeasible since between 75 to 90% of the energy and resources invested in cattle is lost in the animal’s body maintenance and manure production [4]. In addition, it is estimated that the production of 200 g of beef involves the expenditure of 792 L of drinking water, 4 kg of grains for feeding, the deforestation of 6.6 m^2^, and the emission of 50 kg of CO_2_ into the atmosphere [5].

In the world, although there are no global data on followers of meat-free diets, data on vegetarianism show significant numbers in Asia (19% of the population), Africa (16%), South and Central America (8%) and North America (6%) [6]. Furthermore, the number of adherents to diets that remove all or part of meat or meat products is continuously growing [7]. Moreover, given the influence of food on the social interactions of human beings, the search for plant-based meat substitutes is also increasing [3,8]. Therefore, this population needs products that replace meat and its technical and nutritional aspects.

Typically, plant-based meat substitutes consist of products based on a mix of legumes and cereals, using different technologies depending on the final product characteristics, added (or not) by food additives to improve flavor, texture, and appearance [9].

However, several questions are raised about the nutritional quality of these products. Given the objective of complete meat replacement, these plant-based products must have similar or better nutritional quality in the composition and amount of nutrients [10]. In addition, potential health problems related to the additives used to mimic the sensory characteristics of meats are commonly observed in studies [3,11]. In addition, a possible heterogeneity in the nutritional composition of these meat substitutes is expected because of different matrices combinations, making it difficult for consumers to choose the best choice from a nutritional point of view.

In this sense, the objective of this review is to compile and analyze different plant-based meat substitutes (including substitutes for chicken, seafood, and pork) mapped by studies carried out in different countries and, from that, provide better information to consumers to facilitate their understanding of the market.

## 2. Materials and Methods

An integrative literature review was performed with a systematic approach for the best scientific rigor. The search phase for this integrative review was performed according to the Preferred Reporting Items for Systematic Reviews and Meta-Analyses (PRISMA) Checklist [12].

### 2.1. Inclusion and Exclusion Criteria

Only experimental studies related to the quantitative determination of nutrients and ingredients of commercial vegan meat substitutes that imitate products of animal origin were included. Studies with analysis of meat substitutes (including chicken, fish, and pork) were included. Studies categorized as reviews, letters, conference abstracts, case reports, brief communications, and books were excluded from the review; studies that did not quantitatively analyze the nutrients or ingredients in vegan products that seek to mimic products of animal origin were also excluded.

### 2.2. Information Sources

Adapted and individual search strategies were developed for six databases: PubMed, EMBASE, Scopus, Science Direct, Web of Science, and gray literature (Google^®^ Scholar). Patents were searched using the Google Patent^®^ tool. The last search was performed on 1st September 2022. In addition, reference lists of included articles were examined for possible studies not retrieved before.

### 2.3. Search Strategy

At this stage, combined or isolated keywords were used in all the databases in English, and the necessary adaptations were made in each database. The keywords were the following: “Product”, “vegan”, “substitutes”, “meat”, “beef”, “chicken”, “pork”, “plant”, “based”, “commercialized”, “commercial”, “sold”. Endnote Web^®^ and Rayyan Web^®^ software were used to manage bibliographic references.

### 2.4. Study Selection

**Figure 1:**
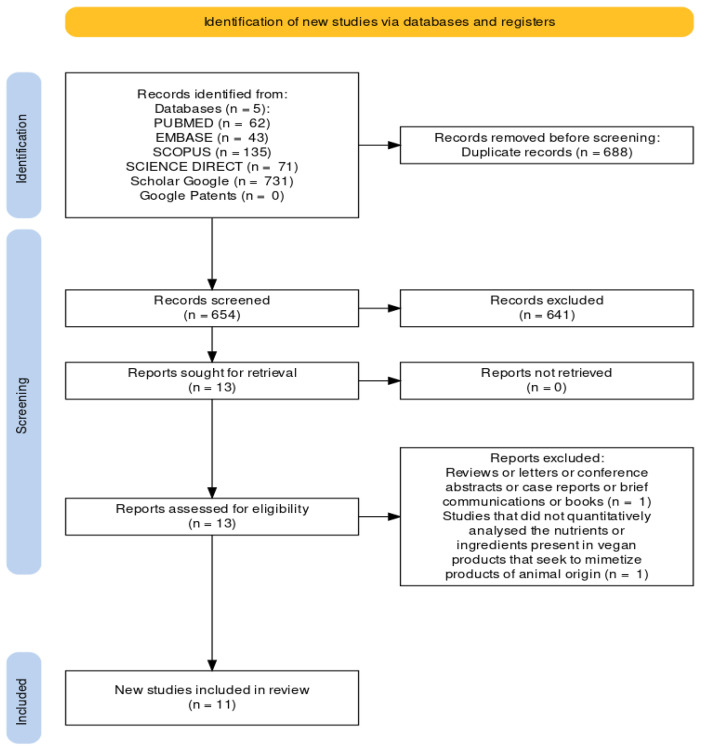
Flow diagram of the literature search and selection phases adapted from PRISMA guidelines [13].

The selection of studies was performed in two stages. At first, two reviewers (B.R. and M.L.T.) independently analyzed the titles and abstracts of all references identified and available in the analyzed databases. Articles that did not meet the inclusion criteria were discarded. Then, after decisions were made by the first (B.R.) and second (M.L.T.) reviewers, a third reviewer (D.d.C.M.) analyzed possible disagreements and determined the potential inclusion or exclusion of the articles. In phase 2, the same reviewers (B.R. and M.L.T.) applied the eligibility criteria to the full texts of the selected articles. In cases of disagreement, the third reviewer (D.d.C.M.) was consulted to resolve disagreements. In addition, two experts (R.B.A.B. and R.P.Z.) on the subject were available to resolve disagreements that could not be dealt with by the third reviewer (D.d.C.M.) and for the inclusion of full texts deemed relevant. The final decision on the articles comprising the sample was made based on the full texts. The flow diagram of the literature search and selection criteria is shown in Figure 1.

### 2.5. Data Collection

The following data were collected from the included works: authors and year of publication, country of study, source of information of the nutritional composition analysis (label or laboratory analysis of chemical composition), the nutritional composition of the products (energy, carbohydrates, sugars, protein, dietary fiber, total fat, saturated fat, sodium, iron, zinc, and vitamin B12). When available, the main ingredients used in the studied products were collected. The complete table with all collected results is available in Table S1 (Supplementary File). Different meat substitutes were grouped into different categories to evaluate the nutritional composition better. The categories and their components are listed in Table 1.

The collected data were synthesized in tables using Microsoft Excel^®^ software (Santa Monica, CA, USA, 2022). Calibration exercises were performed with the designated reviewers (B.R., M.L.T., and D.d.C.M.) to ensure the consistency of the information collected.

### 2.6. Data Classification and Statistical Analysis

The nutritional composition of the collected meat substitutes from included studies was categorized in grams (g) for carbohydrates, protein, dietary fiber, total fat, and saturated fat. Iron and zinc were collected in milligrams (mg) and vitamin B12 in micrograms (mcg). In studies where energy was described as kilojoules (Kj), their respective values were converted to kilocalories (Kcal), using the conversion factor of 4,184 (1 Kcal = 4184 Kj). In products where only the salt (g) content was available, its value was converted to sodium (mg), considering each gram of salt respective to 400 mg of sodium.

The median, maximum, and minimum values of the nutritional composition of meat substitutes were calculated. For comparison purposes, animal-based equivalent nutritional data was collected from the USDA food composition table [14]. The median, maximum, and minimum values of the available products for each corresponding category of vegan products were also calculated. Microsoft Excel^®^ software (Santa Monica, CA, USA, 2022) was used in this stage.

A scatterplot matrix was generated based on the nutritional values collected for each product category. For graphical visualization, a word cloud was generated based on the frequencies of the implemented ingredients on included samples, given that higher frequencies are represented with more prominent words [15]

## 3. Results

In all electronic databases, we identified 654 articles. We did not find a registered patent for meat substitutes. In Phase 1, we selected 13 articles for their potential interest. In Phase 2, two articles were excluded for not meeting the specified criteria. Our experts did not include additional articles. Therefore, 11 articles were eligible for a complete reading. All of these met the eligibility criteria, and all the included studies were published between 2019 and 2022.

### 3.1. Studies General Characteristics

A total of 10 countries published studies regarding the nutritional value of meat substitutes around the world: Denmark [16] (n = 1; 9.09%); USA [17,18] (n = 2; 18.18%); Spain [19] (n = 1; 9.09%); Latvia [20] (n = 1; 9.09%); Italy [21] (n = 1; 9.09%); Brazil [22] (n = 1; 9.09%); Australia [23] (n = 1; 9.09%); Sweden [24] (n = 1; 9.09%); UK [25] (n = 1; 9.09%) and Norway [26] (n = 1; 9.09%).

From all included studies, 64% (n = 7) studied only the nutritional composition of meat substitutes [16,17,18,19,24,25,26]. The remaining four studies (36%) analyzed nutrients and ingredients [20,21,22,23]. Only one study [16] performed chemical analysis to obtain the nutritional value of analyzed meat substitutes. The remaining studies (n = 10; 91%) utilized food labels as their information source.

### 3.2. Meat Substitutes Samples Characteristics

Regarding the categories of most frequently included meat substitutes, 54.54% (n = 6) of the studies included “burgers” in their samples [18,21,22,23,24,25]; 54.54% (n = 6) of the studies included “minced” [16,17,22,23,24,25]; 45.45% (n = 5) of the studies included “sausages” [16,22,23,24,25]; 36.36% (n = 4) of the studies included “meat balls” [16,21,24,25]; and “cold cuts” [16,21,22,24].

In lesser frequency, 27.27% (n = 3) analyzed “seafood” [19,22,23], “chicken cutlets” [22,23,25], and “Chicken nuggets” [22,24,25]; 18.18% (n = 2) of the studies evaluated “pieces” [16,24] and “various” [20,26].

The categories “Cutlets”, “Others” and “Schnitzel” were present in only one study each [21,23,24]. The collected nutritional composition for studied meat substitutes and their respective medians, maximum and minimum values are in Table 2. Table 2 also presents the nutritional composition of animal-based counterparts. The complete composition of the analyzed meat substitutes of the included studies, by category of sample, is available in Table S2 (Supplementary File).

Regarding meat substitutes and animal-based meat, higher energy values were found among the samples of “Chicken nuggets”. In contrast, in the meat substitute groups, lower values were present in the “Minced” category, while in the animal protein, lower values were found in “Pieces” (Table 2). Regarding the carbohydrate concentration in meat substitutes, higher values were shown in the “Seafood” category, whereas in “Cold cuts”, the values for this nutrient are the lowest (Table 2). In animal-based protein products, higher values were found among the “Chicken Nuggets” samples.

In the vegan meat substitutes, the highest values for sugar were found among the “Others” samples. At the same time, categories such as “Chicken cutlets” and “Chicken nuggets” presented less than 1 g of sugar among all samples. Considering animal-protein equivalents, only “Meatballs” presented some amount of sugar.

The protein concentration was higher among the plant-based meat substitutes “Pieces” category, and the samples in “Seafood” presented the lowest content for this nutrient. In the animal protein group, samples belonging to the “Cutlets” category presented the highest protein concentration. Dietary fiber was most present in samples of the “Chicken cutlets” category, while most samples of “Seafood” substitutes did not present dietary fiber. Total and saturated fats were more present in samples of the “Various” category, while “Cold cuts” showed the lowest values. The total content of dietary fiber was shallow among animal-based meats, with only samples from “Meat balls”, “Chicken nuggets” and “Schnitzel” containing this compound in their composition.

Higher values were found in the “Various” samples regarding sodium content, but not all studies provided sodium values for their included samples. In animal-based products, the highest concentration of sodium was found among samples from the “Cold cuts” category. Furthermore, most studies did not analyze iron, zinc, and vitamin B12, since these nutrients are not mandatory on food labels.

**Figure 2:**
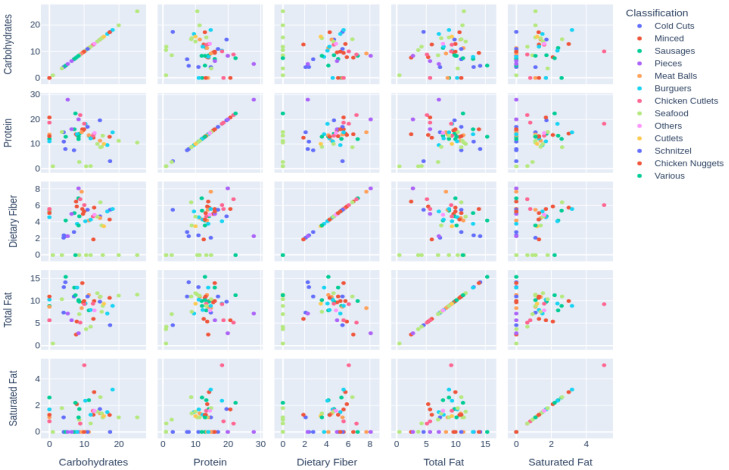
Scatterplot exposing the proportions for pairs of analyzed nutrients for each meat substitute category. Numbers’ units are represented in g/100 g.

A scatterplot related to the proportion of analyzed pairs of nutrients present in each meat substitute for 100 g of the product is available in Figure 2.

Regarding the proportion of carbohydrates and protein, “Seafood” presents more carbohydrates concerning its protein content, whereas “Pieces” present more protein than carbohydrates. As for the proportion of carbohydrates and dietary fiber, “Seafood” presented the lowest values, while “Pieces” presented the highest values. The proportion of carbohydrates and total fat is higher in “Seafood,” with more carbohydrates than total fat content. In “Various” and “Pieces”, the total fat content is higher in proportion to its carbohydrate concentration. In the “Chicken cutlets” category, the saturated fat ratio is higher than its carbohydrates, while the remaining categories tend to present less saturated fat in proportion to carbohydrates.

Regarding the proportion of protein to carbohydrates, the “Seafood” category presented more carbohydrates than protein, while “Various”, “Chicken nuggets” and “Others” presented more protein than carbohydrates. Considering protein and dietary fiber, “Seafood” presented no values regarding its dietary fiber content. Therefore, this category presented more protein than dietary fiber, while the categories “Pieces” and “Cutlets” presented higher concentrations of dietary fiber than protein. The “Various” category presented the highest values for the proportion of saturated fat to protein, while “Seafood” and “Chicken cutlets” presented the lowest. “Chicken cutlets” presented the highest proportion of saturated fat compared to its protein content, while the other categories had lower values for fat.

“Various” and “Chicken nuggets” categories presented lower fiber proportions than total fat. On the other hand, most samples of “Pieces” presented a higher proportion of dietary fiber than their total fat content. A similar distribution between the proportions of saturated fat and dietary fiber was found in all samples, with all having more dietary fiber than saturated fat, with the exception of one sample from the group of “Chicken Cutlets”.

**Figure 3:**
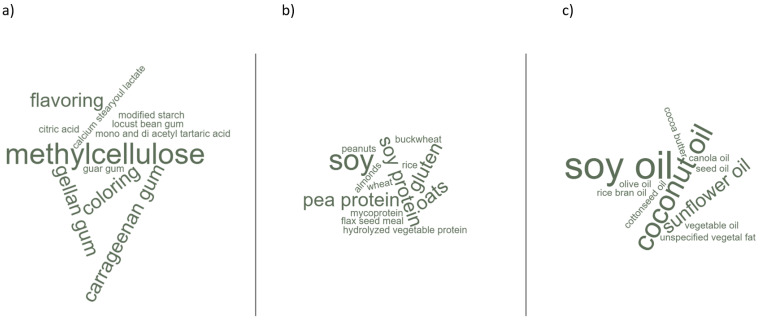
Word cloud generated with the frequencies of implemented ingredients in the included meat substitutes. Higher frequencies are represented with more prominent words in the cloud. (**a**) Food additives; (**b**) Protein sources; (**c**) Fat Sources.

Four studies analyzed the ingredients used as meat substitutes [20,21,22,23] (Table 3). A word cloud generated with the frequencies of the mentioned ingredients is available in Figure 3.

Overall, soy-based ingredients (soybeans, soy protein, isolated soy protein) were the most implemented protein sources in the included meat substitutes, followed by pea-based ingredients (pea protein and peas). Wheat was also present as a protein source in the form of gluten. Pulses are, in general, more frequent than grains.

Thickeners and stabilizers such as methylcellulose and xanthan, gellan, carrageenan, carrageenan, and guar gums were the most frequent additives. As for the fat sources, soy oil was the most used, followed by sunflower, cottonseed, and coconut oils. However, it is important to note that the authors did not describe the ingredients in each meat substitute category. Therefore, it is not possible to provide further information on this subject.

## 4. Discussion

Regarding the included studies, most studies were performed in the USA [17,18]. In this country, the number of adepts of vegetarianism is around 5% of the population, and the number of vegans is about 3% [27]. Furthermore, its plant-based products market is one of the most successful in the world, with a gross revenue of USD 800 Million and a growth projection of almost 25% in size by 2025 [28]. Only one study was produced by researchers in other countries (Denmark, Brazil, Spain, Italy, Australia, Sweden, UK and Norway). In these countries, the prevalence of vegetarianism ranges from 1.4% in Spain to 4% in Brazil [29,30].

Although different prevalence levels of vegetarianism were found within these countries, a common point regarding them is the growth of the plant-based dedicated market. In average, almost 50 million USD were invested in all the cited countries, highlighting the growth of these markets and justifying the presence of the analyzed samples in the included studies [30].

Most studies analyzed only the nutritional composition of food labels. Regarding this analysis, it is important to note that food label laws worldwide present tolerance for discrepancies regarding the actual nutritional value and the values described in food labels [31]. Therefore, a possible limitation regarding the described values is noted [16,17,18,19,24,25,26]. In addition, only four of the included studies analyzed the utilized ingredients in meat substitutes [20,21,22,23]. For better evaluation of the meat substitutes’ overall quality, it is necessary to explore the correspondence of the found nutritional value and the implemented ingredients since, in these products, a large variety of ingredients are commonly used [32].

According to reports, an estimated 720 brands are involved in the meat substitutes market, with around 3000 products already commercialized [33]. In the present study, 1625 samples were collected from the studies, highlighting the need for more studies evaluating the nutritional composition of meat substitutes commercialized worldwide. Furthermore, studies on plant-based meat substitutes were performed in 10 countries, representing only 10.97% of the globe.

In this sense, it is also essential to highlight questions about the production of vegan meat substitutes. Exporting products is one of the alternatives practiced by countries whose industries are not yet fully developed [33]. However, when observing the sustainable development objectives advocated by the United Nations Organization, the local production of inputs is a goal to be achieved [34]. Thus, given the premise that meat substitutes should be more sustainable alternatives than beef, industries in these other countries must be developed to achieve this objective fully.

### 4.1. Energy

In traditional diets, meat concentrates the highest number of calories in large meals such as lunch and dinner [35]. Considering the contribution of these meals as 30–40% of an individual’s total daily energy value, meat typically represents up to 70% of all calories in these meals (250–400 kcal) [36,37]. Different types of meat (Table 2) present on average between 65 and 80% of water, 16 to 22% of proteins, 3 to 13% of fats, and few amounts of vitamins and minerals [38]. In this sense, the high amount of protein and fat contributes to its total energy value [38].

It is commonly observed that plant-based meat substitutes are made from a combination of legumes and cereals, naturally containing more carbohydrates than fats in their composition [22,39]. Thus, meat substitutes tend to have lower energy values than their animal counterparts [22,26]. This characteristic agrees with the characteristics of plant-based diets, whose caloric value tends to be reduced compared to diets with a more ostensible presence of meat, such as the Western diet [40]. In the present review, the values found in the item “Energy” ranged between 170 and 217 kcal, lower than those traditionally provided by meat. Therefore, using meat substitutes can constitute a viable alternative for an energetic reduction in diets, contributing to weight loss and prevention of chronic non-communicable diseases (NCD) such as obesity, type 2 diabetes mellitus, and coronary heart disease [41,42].

Furthermore, it is important to highlight that lower energy values were found in categories whose objective is to mimic in natura meats (Minced, pieces, cold cuts), constituting interesting options for substitution in meals, at least from the energy value point of view. In categories such as “Burgers” and “Chicken nuggets”, higher amounts of calories were found, accordingly to their animal counterparts, constituting plant-based versions of “treats” and “junk foods”.

### 4.2. Carbohydrates and Sugars

Carbohydrates are the most common nutrients in vegetables since they are usually present in their composition of saccharides of the most diverse sizes and complexities, such as starch and polyols [43]. Within the context of meat substitutes, the most frequent ingredients (legumes and cereals) are rich in carbohydrates, fluctuating between 50% and 85% of their proximate composition [38,44]. Therefore, it is expected that meat substitutes present higher values of carbohydrates in comparison with meat, as confirmed in this review, with values between 5.85% and 13.83%. A higher proportion of carbohydrates in vegan products compared with other present nutrients was also found, thus, reinforcing this tendency even more.

In four of the included studies [18,22,23,26], a comparison was made between the carbohydrate values of meat substitutes and their respective animal counterparts. In general, they pointed to significantly higher concentrations of carbohydrates in the plant-based versions, with values ranging from 7–15 g/100 g in plant-based meat substitutes, compared with meat, with 0–3 g/100 g [18,22,23,26]. These values are close to those found by other studies included in this review, demonstrating a higher concentration of carbohydrates in plant-based meat substitutes.

However, despite the greater amount of carbohydrates, this characteristic may not necessarily negatively influence the quality of diets that include meat substitutes. In a study where the effects of a plant-based diet rich in carbohydrates originating from whole grains and legumes and reduced in fat were analyzed, the authors mentioned the effectiveness of this diet in weight loss and better quality of life [43]. Therefore, despite the greater amount of carbohydrates in meat substitutes, since they come from legumes and cereals, the carbohydrate content would not be excessive in a diet in which meat substitutes are included, based on this ingredient alone [43].

The “Seafood” category presented the highest carbohydrate values among the analyzed categories. This is probably to obtain a gelatinous texture (like fish), given the inherent characteristic of carbohydrates to form stable gels with water and heating, in a physicochemical process called gelatinization [19,45]. The “Cold cuts” category had the lowest amount of carbohydrates. This category consists of substitutes for meats used in sandwiches and snacks, such as hams, salami, and other foods from the same class, whose nature is more protein-based and usually presents fewer carbohydrate amounts [16,21,22,24].

Sugars were present in smaller amounts in the samples analyzed by the studies. Commonly in plant-based substitutes, sugars are found most prominently in dairy substitutes, as they act as stabilizers and thickeners and try to mimic the characteristic sweetness of another disaccharide, lactose, which is present in dairy products [46]. Naturally, meats have negligible concentrations of mono and disaccharides and are not foods with a sweet taste in general. In this sense, the low use of this ingredient in plant-based meat substitutes is expected [38]. The category with the highest amount of sugars was “Seafood”, an ingredient possibly used to obtain some technical characteristic unrelated to flavor. However, the studies did not explore this ingredient and its respective industrial characteristics [19,22,23].

### 4.3. Dietary Fiber

Dietary fibers are provided exclusively from foods of plant origin, and their applications are manifold from the point of view of health maintenance and technological improvement of meat substitutes [22]. In the context of health aspects, dietary fibers contribute in maintaining health by favoring good intestinal functioning and collaborating in maintaining healthy intestinal microbiota [47,48]. In addition, during the digestive process, soluble and insoluble dietary fibers in the intestinal lumen form bulky and viscous molecular complexes that reduce the rate of absorption of carbohydrates, saturated fats, and cholesterol, thus helping to maintain a healthy weight and prevent NCD [47,48].

In general, studies describe that the dietary pattern most practiced in Western countries consists of the consumption of industrialized foods of animal origin, fattier and with a lower amount of dietary fiber [49,50] In this sense, this dietary pattern is associated not only with increases in the prevalence of NCDs, but it also causes changes in the intestinal microbiota, permitting the disordered growth of gram-positive bacteria, especially those of the *Clostridium* and *Proteobacteria* class, whose studies point to a relationship with brain health, among other negative changes [49,50].

Meat commonly does not have dietary fiber in its composition, contrary to what was evidenced by the meat substitutes analyzed in this review, whose values ranged from 0 to 5.84 g/100 g. In animal-based meats with dietary fiber (Chicken nuggets and Schinitzel), this value is due to the addition of cereals to bread the meats. Current dietary reference intakes (DRIs) recommend daily fiber consumption of 30–35 g for men and 25–32 g for adult women. In this way, a single 100 g serving of meat substitute (Chicken cutlets) can contribute about 16.68% of the recommended daily value [51] Thus, meat substitutes may be interesting alternatives for increasing dietary fiber consumption, especially in Western diets, where fiber consumption is reduced.

Regarding the technological and sensorial characteristics of the fibers, they can retain water in products in which they are present, favoring characteristics such as texture and resistance to breakage, characteristics also present in meats [47]. However, the excessive use of dietary fibers in these products results in negative characteristics in the same way, resulting in more rigid products requiring excessive chewing [22]. Therefore, even based on plant-based matrices, which could provide even greater quantities than those found, the excessive use of fiber in meat substitutes would impair their palatability and consequently, their commercialization.

Furthermore, dietary fibers’ characteristic hygroscopicity also influences cooking oil retention. Thus, in the case of raw or pre-cooked meat substitutes, which require the use of cooking methods such as grilling or frying, this may result in an amount of fat even higher than described on the labels.

### 4.4. Protein

In the Western diet, proteins are mainly supplied by foods of animal origin, in greater quantity by meats, followed by eggs and dairy products [21]. In addition to cultural and environmental subjects, it is important to highlight that protein stands out among the primary nutrients provided by meat, reaching almost 22% of its composition [52]. On the other hand, plant-based products commonly have lower amounts of protein, with values ranging between 0.3 and 11%, in the case of legumes, which contain the highest amount of protein [53]. In this sense, protein intake is one of the main concerns in eating meatless diets, demanding attention from health professionals and the elaboration of public health policies [54]. Meat substitutes are usually made from legumes, especially soy, peas, chickpeas, beans, and some cereals such as wheat (gluten) and oats [20,21,22,23]. As evidenced by the studies included in this review, soy and its derivatives constituted the main protein source in meat substitutes (Figure 3).

Soybean stands out among legumes for several reasons, firstly for its economic value. Currently, the soy market has an export value estimated at around 27.39 billion dollars. Its production totals about 53 million metric tons on the planet, and it is one of the primary commodities exported by countries such as China, Mexico, and the European Union [55]. In addition, this legume stands out for its protein value (about 38% of its proximate composition) [56]. However, it is essential to note that during the cooking process, soybeans absorb water and swell to around 2–3 times their original size [56]. In this sense, its nutritional density is diluted; therefore, larger portions are needed to obtain protein values comparable to what is provided to animal-based meat in 100 g. It is noted that multiple technologies can be used for better technological and sensory use of this legume. One of the most used technological processes in the soy industry is hydrostatic extrusion, which consists of an assisted grinding and friction heating process, which results in one of the most used products in the meat substitute industry, textured soy protein [56,57].

Textured soy protein is an ingredient whose texture and appearance resemble meat, and its physicochemical structure and capability of absorbing liquids and flavors enable the use of diverse ingredients for flavoring, including food additives whose composition is intended to mimic the flavor, aroma, and color of the meat [22,57]. Nevertheless, the defatted, dehydrated, and isolated soy protein extract also provides interesting sensory and technological characteristics in manufacturing meat substitutes [57]. The same technologies can be used in other legumes, such as peas, which appear as protein alternatives for the formulation of soy-free meat substitutes, as part of the population avoids soy due to health problems or personal preferences [58,59]. Wheat gluten is also one of the most used ingredients in meat substitutes, given its protein composition with viscoelastic capacities that simultaneously contribute to the nutritional composition of these products and to sensory and physicochemical characteristics (elasticity, tenacity and resistance) [60,61].

Since the meat substitutes analyzed are mainly composed of legumes and gluten, their nutritional composition is proportionally richer in protein in an attempt to fully replace meat of animal origin.

In the present review, the median values referring to the protein quantity of meat substitutes range between 8.9 g/100 g (Seafood) and 20 g/100 g (Pieces). However, analyzing the mean values of the same nutrient present in beef, the average value is 25 g/100 g [62], demonstrating that the protein value offered by meat substitutes is still lower than that usually offered by meat, especially in comparison with their animal-based equivalents (Table 2). In the case of plant-based substitutes for chicken, the median value (18.77 g/100 g, “Chicken Cutlets”) is also lower than that offered by its animal-derived counterpart (20 g/100 g), reinforcing the need to develop plant-based alternatives with a higher amount of protein [62]. The same analysis is also verified when analyzing the other included categories.

Another issue involving the use of plant proteins as substitutes for their animal counterparts is their bioavailability. There are several methodologies to assess protein quality, such as the PDCAAs (protein digestibility-corrected amino acid scores) and the DIAAS (digestible indispensable amino acid scores), the latter being the most recent and most suitable for analyzing the bioavailability of plant proteins [63,64]. In general, plant proteins have a reduced amount of digestible essential amino acids, especially compared to highly digestible animal proteins, such as ovalbumin in eggs and whey proteins from cow’s milk [63]. However, this limitation can be circumvented by combining two or more plant proteins, as they have different digestible essential amino acid values. Some have greater amounts than others in specific amino acids, such as branched-chain amino acids [63]. In this sense, since many meat substitutes combine at least one legume and one cereal, there is a possibility that they offer a better-quality protein combination when compared to portions of isolated legumes. However, more in vivo studies are needed to confirm this hypothesis.

### 4.5. Total and Saturated Fat

The total and saturated fats levels constitute one of the biggest problems concerning meat consumption. Depending on the type of cut used and the breed and diet of the animal, the meat fat content can vary between 1 and 28 g/100 g [35,37]. Values for fat concentration may also vary according to the implemented cooking method.

Currently, the DRIs do not indicate maximum values of total fat consumption by age group. However, it is known that their energy contribution should be between 20–35% of the daily value ingested [51,65]. In this sense, it appears that a portion of the category with the highest total fat content (Pieces) contributes about 4.8% of the total recommended energy value for this nutrient in a diet of 2000 kcal (10.75 g/100 g, 96.75 kcal). In comparison with a typical cut of beef, it appears that it contributes 4.9% of the recommended daily intake (10.9 g/100 g, 98 kcal). This value is close to the meat substitute with a higher total fat content [62]. However, it is important to consider the variation in fat contents between the different categories of meat substitutes, which, as well as cuts of meat of animal origin, also have alternatives with lower fat contents.

Another important point to consider is the sources of fat used in meat substitutes. The verified results show that the meat substitutes mostly used vegetable oils, such as soybean, sunflower, olive, and cottonseed oils [20,21,22,23]. Concerning the composition of these oils, they have primarily poly and mono-unsaturated fatty acids, whose metabolic effect is different from that of saturated fat, found in greater amounts in the meat [66,67].

As sources of omega-6 fatty acids, these oils mainly contribute to several organic functions, such as the structure and fluidity of the plasma membrane of human body cells [68,69] However, these same fatty acids, when consumed in excess, act in the synthesis of pro-inflammatory cytokines, in addition to data indicating that the world consumption of omega-6 fatty acids is excessive, given their presence both at home and in industrialized foods of plant and animal origin [68,69]. In this sense, even though they are composed of vegetable oils, the fat contents found in meat substitutes indicate that they should not be consumed excessively.

Another issue regarding the fat content of meat substitutes is the possible absence of omega-3 fatty acids, specifically in the “Seafood” category. Eicosapentaenoic acid (EPA), and docosahexaenoic acid (DHA) are fatty acids from the class of omega-3 found in animal-based seafood [70]. As for vegetable sources, omega-3 is found not as EPA or DHA, but as Alpha-linoleic acid (ALA), which can be converted into both EPA and DHA through a metabolic pathway. In this sense, vegetable sources such as flaxseeds, chia seeds, and seaweed are known sources of ALA, so it is preferable that these ingredients are implemented in plant-based seafood to provide comparable amounts of omega-3 fatty acids [70,71]. Furthermore, a thorough analysis of the implemented ingredients in plant-based seafood substitutes is needed to quantify the amounts of ALA.

Regarding the amount of saturated fat presented by the analyzed meat substitutes, the maximum value of 1.65 g/100 g was found in the “Various” category, with emphasis on the “Burger” categories (1.6 g/100 g) and “Chicken nugget” (1.28 g/100 g). In meat substitutes, saturated fat sources typically consist of fats from coconut and palm, plant sources that behave similarly to those of animal origin [22,23]. However, compared to a typical cut of beef, which has between 3 to 9 g of saturated fat per 100 g serving, meat substitutes still have lower values, thus constituting better options [62].

In addition to the characteristics related to the nutritional quality of foods, fat also contributes to products’ sensory characteristics, such as lubricity, palatability, aftertaste and shelf life. All characteristics are desirable for food marketing and acceptance [72]. Therefore, despite the lower amount of natural fat in products of plant origin, the manufacture of fat-free meat substitutes is unfeasible, as this would affect their sensory characteristics, making these products undesirable.

### 4.6. Sodium

Excessive sodium consumption is one of the biggest public health problems today, mainly given its ostensible use in industrialized products, as in the case with plant-based meat substitutes [72,73]. Naturally, meat has reduced sodium content in its composition. In addition, it has nitrogenous compounds responsible for flavor development and collaboration in flavor development chemical reactions, such as the Maillard reaction [74]. The biggest problem lies in processed meats, such as hamburgers, ham and sausages, which have high sodium contents, prolonging their shelf life and palatability [72,73].

The tendency to use sodium is verified in most meat substitutes, possibly in an attempt to use flavor, given the absence of natural compounds related to this aspect in products of plant origin [11,24,41].

In this review, sodium values between 210 mg (Cold cuts) and 900 mg (Various) were found, thus demonstrating a trend toward excess sodium in meat substitutes.

In the studies where implemented ingredients were analyzed, it is important to note that salt (or sodium) was absent. Probably, salt was added only on the nutritional label, in the form of salt or sodium, or the analysis was not performed. There is also a possibility that some of these products are commercialized as salt-free options for further seasoning. In this manner, a possible limitation regarding this absence is noted.

Currently, the World Health Organization recommends a daily intake of 2300 mg of sodium per day. A 100 g serving of some categories of meat substitutes can contribute up to 39% of the total recommended daily value [75].

Behaviors such as using natural seasonings, herbs, and sodium-free condiments can be alternatives for reducing sodium in meals, an attitude that is necessary for several diets, especially those aimed at controlling cardiovascular diseases [76,77]. Thus, in the current model of commercialization of meat substitutes, with built-in amounts of sodium, it is impossible to use strategies to formulate healthier meals, demonstrating a gap and a necessary improvement in the formulation of these products.

### 4.7. Iron, Zinc, and Vitamin B12

Iron is one of the minerals provided by meat and adapting the consumption of this micronutrient in meatless diets is a well-known challenge [78,79]. Traditionally, adherents of vegetarian diets tend to consume lower amounts of iron, not only because this nutrient is present in lesser amounts in plant-based foods but also because of the reduced consumption of source foods, such as dark green vegetables [79].

In addition, another problem is found in the chemical structure of the iron supplied by vegetables, whose electronic charge (+3) lacks specific intestinal receptors. The hemic iron present in meat of animal origin, in contrast, has an electronic charge is +2 and a specific intestinal transporter, favoring its metabolism [78,79].

Current DRIs recommend daily values of iron intake between 8 and 10 mg, depending on the age and gender of the person [51]. In the context of the meat substitutes analyzed, specimens of the “Minced” category presented about 10 mg of iron per 100 g of products [17], fulfilling fully or mainly with the daily need for this element. However, it is important to highlight that iron is not an element of mandatory declaration on food labels according to the legislation in force in several countries that produced the studies included in this review. Since most studies used food labels as a source of information, a limitation of this review is the lack of information on this mineral. The same problem occurs regarding zinc and vitamin B12, whose declaration is optional, and not present in most of the labels analyzed by the studies.

Regarding meat-free diets, it is important to highlight that legumes and cereals are the main sources of iron and zinc. Thus, since these ingredients are the most implemented in the analyzed meat substitutes, there is a possibility that these nutrients are present in adequate amounts. However, future studies with laboratory analysis are necessary to verify it [32,80].

Vitamin B12 is produced by microorganisms and is available for metabolization into products of animal origin from the bioaccumulation process through livestock feed [81]. In this sense, it is important to highlight that foods of animal origin are exclusive sources of this vitamin, and in the context of meat-free diets, they must be supplemented or acquired through fortified foods [81].

Given the absence of this vitamin in foods of vegetable origin, it is common practice to fortify meat substitutes with vitamin B12. However, given this information’s absence in the studies, it is impossible to analyze the contribution of this fortification in meat substitutes [81].

### 4.8. Food Additives

Food additives are classified as substances that are not nutrients but are used in foods to improve its technical and sensory characteristics [82]. In meat substitutes, one food additive classification that stands out is flavorings.

Flavoring agents can be of natural or synthetic origin, and their purpose is to impart flavor to foods. In the case of meat substitutes, the characteristic flavor of the meat is to be mimicked [82]. For example, beef has nitrogenous bases in its composition that give it a characteristic flavor, thus requiring little additional seasoning. In the case of meat substitutes, given the absence of these compounds, the use of flavorings is necessary, given the objective of these products to simulate the traditional version of meat [22,38]. In the case of the analyzed meat substitutes, these were found in all samples that included the analysis of the ingredient in their scopes [20,21,22,23]. However, these may also be present in samples for which this analysis was not performed.

Another subject regarding the flavoring of meat substitutes is the absence of endogenous metabolic pathways that directly influence the meat’s flavors. For example, postmortem phenomenon such as rigor mortis and fermentation in controlled conditions interferes with meats’ pH, therefore, satisfactorily altering its flavor [83,84]. In this sense, artificial flavoring is needed to provide similar flavor in meat substitutes, or even further studies to evaluate the possibility of replicating such processes in plant-based matrices.

Hydrocolloids are also used in meat substitutes, such as methylcellulose and gums from diverse origins. Hydrocolloids consist of carbohydrate molecules of microbiological or plant origin, which can form gels that improve the texture, strength and tenacity of products in which they are present [85]. In the case of methylcellulose, it can remain in a solid state after gelatinization, and its appearance resembles fat complexes, commonly present in beef analogs [85].

From a nutritional point of view, hydrocolloids characterize substitutes for dietary fiber since, after hydration, they form complex and viscous molecular structures that can delay the absorption of carbohydrates, such as dietary fibers and fat [85,86]. Thus, its presence can be beneficial given the high value of carbohydrates present in meat substitutes.

## 5. Conclusions

This review evaluated the nutritional compositions of meat substitutes commercialized worldwide. Most studies used food labels as their information source, and few analyzed the nutritional composition and implemented ingredients in meat substitutes. The results showed that meat substitutes are not like meat, commonly presenting lower energy values and higher amounts of carbohydrates and dietary fiber, given their plant-based origin. Furthermore, protein values varied according to the meat substitute category, with some presenting a higher concentration than others, more specifically in substitutes for bovine meat. In meat substitutes, the proportion of carbohydrates is higher than the protein concentration in most samples except the chicken substitutes. Furthermore, meat substitutes presented similar total and saturated fat content compared to animal-based counterparts. Ingredients such as soy, pea, and wheat were the main protein sources utilized in meat substitutes, while vegetable oils were represented as their fat source. Methylcellulose, various gums, and flavorings were the most frequently used food additives.

In general, meat substitutes presented high concentrations of sodium, possibly contributing to excessive sodium intake, highlighting the need for developing sodium-free alternatives. The concentrations of Iron, Zinc, and Vitamin B12 were not described by most of the included samples, possibly because these nutrients do not require mandatory declaration on food labels, thus constituting a limitation of this study. Further studies are needed to develop meat substitutes with better nutritional compositions, fulfilling the need for equivalent substitutes for animal-based meat. In addition, studies evaluating the dietary impact of total replacement with the analyzed meat substitutes are needed to better comprehension of this subject in the long term.

A limitation of the study is related to the samples’ nutritional data statistical analysis. In the preliminary statistical analysis phase of the study, the standard deviations for the nutritional values of meat substitutes were too far from the mean values, impairing our best analysis.

## Figures and Tables

**Table 1:** Developed categories and their respective components.

Burgers	*Bovine meat burgers, “beef” burgers, red meat burgers*
Meat Balls	*Red beef minced balls*
Minced	*Bovine meat minced beef*
Pieces	*Red meat fillets, medallions, scallops*
Chicken Cutlets	*Chicken wings, chicken breast, chicken hamburgers*
Chicken Nuggets	*Breaded chicken, breaded chicken balls,*
Cold Cuts	*Hams, bologna, turkey breast*
Sausages	*Sausages, pepperoni*
Seafood	*Fish cakes, canned fish, tuna, shrimps, calamari, fish fingers, fish sticks, salmon, caviar, and fillet*
Cutlets	*Bovine meat cutlets*
Others	*“Vegan Roast,” “Bacon-Style Rashers,” and “Polony.”*
Schnitzel	*German chicken schnitzel*
Various	*Meat substitutes without discrimination about the category of the product.*

**Table 2:** Nutritional composition of meat products and their vegan substitutes with their medians, maximum and minimum values in 100 g of the product.

Type of Product	Energy (Kcal)	Carbohydrates (g)	Sugars (g)	Protein (g)	Dietary Fiber (g)	Total fat (g)	Saturated Fat (g)	Sodium (mg)	Iron (mg)	Zinc (mg)	Vitamin B12 (mcg)
Vegan Burgers	196 (216–175)	11.13 (18.22–0)	0.94 (3.4–0)	13.15 (18.21–9.6)	4.45 (5.6–3.8)	9.17 (13–7.2)	1.6 (3.2–0)	410 (440–372)	0.039 (3.6–0)	0 (0)	0 (0)
Meat Burgers	259 (281, 189)	0 (0)	0 (0)	17.6 (18.6, 14)	0 (0)	20 (22, 16)	12 (15, 8)	93 (113, 21)	1.69 (2.83, 0.67)	2 (3,1)	2.5 (3, 0.67)
Vegan Meat Balls	187 (211–171)	10.32(14.6–0)	1.15 (1.8–0)	13.75 (14.8–11.4)	5 (7.7–4.2)	9.8 (11.35–8.4)	0.55 (1.4–0)	430 (440–0)	0 (2.1)	0 (0)	0 (0.38)
Meat Balls	235 (301, 211)	8.24 (11, 2.8)	3.53 (4.0, 1.1)	16.5 (17, 15)	2.4 (2.5, 1.3)	15.3 (19, 11.2)	8 (9, 4)	682 (711, 233)	2.1 (3.1, 1.1)	2.3 (2.6, 0.88)	2.2 (3.5, 0.8)
Vegan Minced	170 (230–109)	8.95 (12.91–0)	0.2 (1.9–0)	14.9 (20.8–12.6)	5.68 (14–2.5)	5.85 (14–2.5)	0.65 (3.01–0)	272.50 (572.96–0)	0 (10)	0 (0.7)	0 (0)
Minced	183 (296, 112)	0 (0)	0 (0)	19 (22, 13)	0 (0)	8 (12, 5)	6 (10, 3)	119 (221, 45)	2.3 (2.9, 1)	3 (3.4, 0.64)	2.4 (2.9, 0.87)
Vegan Pieces	171 (198–136)	6.4 (8.4–5.3)	0.7 (1.1–0.2)	20 (28–16.05)	5 (8.1–2.3)	5.7 (7.2–2.8)	0 (0)	0 (445)	0	0 (3)	0
Pieces	125 (180, 88)	0 (1, 0)	0 (0)	24 (28, 15)	0 (0)	16 (21, 10)	10 (12, 2)	244 (321, 88)	2.2 (3, 1)	3.8 (4.4, 0.88)	2 (2.4, 0.66)
Vegan Chicken Cutlets	180 (201–161)	9.48 (12.6–0)	0 (0)	18.47 (21.77–13.4)	5.84 (6.79–4.7)	7.49 (9.4–5.17)	1.2 (5.04–0.63)	483.33 (520–372.38)	0 (4.8)	0 (0)	0 (0)
Chicken Cutlets	184 (211, 147)	0 (0)	0	24 (25, 18)	0 (0)	9 (10, 5.6)	4 (6, 3)	98 (112, 33)	1.09 (2, 0.88)	2.07 (3.23, 1.66)	0.38 (0.5, 0)
Vegan Chicken Nuggets	217 (233–216)	10 (17.38–0)	0 (1.1)	13.2 (16–12.97)	5.1 (5.3–4.32)	10.7 (11–10)	1.28 (1.3–0)	480 (499.62–420)	0 (2.1)	0	0 (0.38)
Chicken Nuggets	326 (411, 281)	14.3 (15, 9.1)	0 (0)	16.5 (18.1, 12)	1 (1, 0)	22.6 (24, 18)	16 (17, 14)	708 (881, 637)	0.62 (0.8, 0.55)	0.61 (0.9, 0.55)	0.33 (0.38, 0.21)
Vegan Cold Cuts	173 (251–142)	5.85 (17.5–4.1)	1.1 (5.9–0)	9.5 (19.64–3.1)	2.6 (5.5–2.1)	10.42 (14.2–4.6)	0 (1.73)	210 (840–0)	0 (0)	0 (0)	0 (0)
Cold Cuts	221 (289, 194)	0 (1.4, 0)	0 (0)	16.5 (19, 10)	0 (0)	16.7 (17, 14.6)	14 (18, 9.5)	1190 (1300, 685)	0.83 (0.9, 0.5)	1.94 (2.3, 1.77)	0.92 (1.4, 0.66)
Vegan Sausages	182 (212–136)	7.8 (11.4–0)	1.15 (2.2–0)	13.2 (16–12)	4.90 (6.9–4.2)	9.925 (15.4–7.9)	0.865 (2.6–0)	493.50 (572–0)	0 (3.4)	0 (0)	0 (1.25)
Sausages	309 (401, 299)	0.94 (1.4, 0.2)	0.24 (0.24, 0)	12 (14.1, 9.3)	0 (0)	28.2 (30, 21.1)	19 (21, 10)	827 (900, 582)	0.59 (1, 0.33)	1.31 (1.9, 0.76)	0.66 (0.71, 0.46)
Vegan Seafood	194 (243–13)	13.83 (25.35–1)	0.8 (3.3–0)	8.9 (14.9–1)	0 (6.41–0)	8.9 (11.75–0.75)	1.1 (2.63–0)	420 (1360–136)	0 (0)	0 (0)	0 (0)
Seafood	101 (214, 87)	0.2 (0.3, 0)	0 (0)	24 (26, 12)	0 (0)	14 (18, 0.28)	9 (10, 4,8)	111 (138, 47)	0.51 (1.1, 0.23)	1.64 (1.8, 0.7)	0.2 (0.3, 0)
Vegan Cutlets	196 *	15.7 *	0.9 *	10.1 *	3.5 *	9.4 *	1.2 *	420 *	0 *	0 *	0 *
Cutlets	151 (183, 99)	0 (0)	0 (0)	31.9 (35, 19)	0 (0)	4.64 (4.9, 2.63)	1.6 (2, 0.5)	88 (100, 65)	1.39 (1.8, 0)	3.29 (4, 1.1)	2.72 (3.1, 0.88)
Vegan Others	185 *	13 *	3.2 *	14.5 *	4.9 *	7.9 *	1.6 *	568 *	3.2 *	0 *	0 *
Others	233 (311, 189)	0 (0)	0 (0)	15.3 (18, 9)	0 (0)	16.3 (18, 14.3)	3.8 (4.4, 1)	724 (800, 63)	2.06 (2.88, 0.91)	6.08 (7, 4)	2.38 (3, 1,4)
Vegan Schinitzel	196 *	11 *	1.2 *	17 *	5.5 *	11 *	0 *	440 *	2.1 *	0 *	0.38 *
Schinitzel	211 (284, 93)	6 (8, 4)	0 (0)	17 (19, 11)	0.8 (1.1, 0.6)	14.75 (16, 10)	2.8 (3.1, 2)	500 (550, 480)	4.4 (4.8, 3)	3 (3, 0)	1.6 (2, 1,13)
Vegan Various	214 (228–201)	7.95 (8.4–7.5)	1.45 (1.9–1)	17.7 (22.4–13)	1.8 (3.6–0)	10.75 (11.3–10.2)	1.65 (2.2–1.1)	900 (1200–600)	0 (0)	0 (0)	0 (0)
Various	380 (488, 212)	2 (3, 1)	0 (0)	26 (32, 16)	0 (0)	18 (22, 9.4)	3.6 (5.2, 2.8)	589 (630, 86)	4.8 (5.4, 1.7)	3.33 (5.6, 1.45)	2 (2.88, 0.75)

* Only one study included this category of product; “Vegan Roast,” “Bacon-Style Rashers,” and “Polony.”; Meat substitutes without discrimination about the category of the product.

**Table 3:** Main ingredients in meat substitutes available in the included studies.

Authors	Included Categories	Main Sources of Protein	Main Sources of Fat	Main Food Additives
Curtain et al. [23]	Burgers, Sausages, Minced, Chicken, Cutlets, Seafood, Others,	Soy Protein, pea protein, soybeans, hydrolyzed vegetable protein, mycoprotein, almonds.	Vegetable oil, canola oil, sunflower oil, sunflower kernels, rice bran oil, coconut oil, flax seed meal, cocoa butter, peanuts	N/A
D’Alessandro et al. [21]	Burgers, Cold Cuts, Cutlets, Meat Balls	Soy, Soy derivatives, Rice, Oats and Buckwheat	Seed Oil and Olive Oil	Modified Starch, Citric Acid, Flavouring and Coloring
Mariseva et al. [20]	Various	Soy, Wheat, Starch (Potato and Corn), Pulses, and Oats	N/A	Gellan gum, locust bean gum, guar gum, carrageenan, xanthan gum, methylcellulose, mono and diglycerides of fatty acids, mono and di acetyl tartaric acid, esters of mono and diglycerides, calcium stearoyl lactate
Romão et al. [22]	Burgers, Minced, Chicken Nuggets, Chicken Cutlets, Chicken Cutlets, Seafood, Sausages, Cold Cuts	Soy, Gluten (Wheat), Pea Protein, Isolated Soy, and Pea Proteins	Unspecified vegetal fat, Soy Oil, Sunflower Oil, Cottonseed Oil, Coconut Fat, Coconut Oil	Methylcellulose, Xanthan Gum, Gellan Gum, Carrageenan Gum

## Data Availability

No new data were created or analyzed in this study. Data sharing is not applicable to this article.

## Supplementary Materials

The following supporting information can be downloaded at: https://www.mdpi.com/article/10.3390/foods12030448/s1, Table S1: Full data regarding the information collected in the included studies, Table S2: Full data regarding the nutritional composition of the samples of the included studies.
