# Supplementary material for: Nutritional Profile of Commercialized Plant-Based Meat: An Integrative Review with a Systematic Approach
Source: Foods. 2023 Jan 18;12(3):448. doi: 10.3390/foods12030448 (PMC9914738; doi:10.3390/foods12030448)
Supplement: Supplementary file 1 [file foods-12-00448-s001.zip › foods-2114480-supplementary.pdf]

Table S1 – Full data regarding the information collected in the included studies

AUTHOR	YEAR	COUNTRY	TITLE	INGREDIENTS AND NUTRIENTS?	FOOD LABELS OR CHEMICAL ANALYSIS	TYPE OF PRODUCTS	SAMPLE SIZE	MAIN INGREDIENTS	MAIN FAT SOURCES	MAIN PROTEIN SOURCES	MAIN FOOD ADDITIVES
Svarc et al.,	2021	Denmark	Nutrient content in plant-based protein products intended for food composition databases	Nutrients	Chemical Analysis	Minced; Pieces; Sausages; Minced Balls, Cold Cuts, Seitan	58	N/A	N/A	Minced: (Mycoprotein, Pea Protein, Soy Protein); Pieces (Mycoprotein, Soy Protein); Sausages (Pea Protein, Soy Protein); Cold Cuts (Soy Protein, Pea Protein, Wheat Protein); Seitan (Wheat Protein)	N/A

Mariseva et al.,	2020	Latvia	ASSESSMENT OF INGREDIENTS AND NUTRITIONAL VALUE OF VEGAN PRODUCTS IN LATVIAN MARKET	Ingredients and Nutrients	Food Labels	Beverages, Dairy Alternatives, Meat Substitutes	80	Beverages: Soya, Starch (Potato and Corn) and Oats; Dairy Alternatives: Soya, Wheat and Starch (Potato and Corn); Meat Substitutes: Soya, Wheat, Starch (Potato and Corn), Pulses and Oats	N/A	Soya, Wheat and Pulses	Gellan gum, locust bean gum, guar gum, carrageeans, xanthan gum, methylcellulose, mono and diglycerides of fatty acids, mono and di acetyl tartaric acid, esters of mono and diglycerids, calcium stearyoul lactate

Boukid et al.,	2022	Spain	Seafood alternatives: assessing the nutritional profile of products sold in the global market	Nutrients	Food Labels	Tuna. Shrimps, Calamari, Fish fingers, Fish Sticks, Salmon, Caviar and Fillet	149	N/A	N/A	N/A	N/A
Romão et al.,	2022	Brazil	Are Vegan Alternatives to Meat Products Healthy? A Study on Nutrients and Main Ingredients of Products Commerciali zed in Brazil	Ingredients and Nutrients	Food labels	Hamburgers; Minced Beef; Meatballs; Breaded Chicken; Chicken Hamburgers; Chicken Breast; Canned fish; Fish Cakes; Sausages and Hams	125	Ingredients derived from legumes (soy, peas, chickpeas, and beans) and gluten were the most used in the studied samples	Unspecifie d vegetal fat	Soy Protein, Gluten, Pea Protein, Chickpeas, Beans	Methylcellulose, Xanthan Gum, Guar Gum, Flavouring

Harnack et al.,	2021	USA	Nutrient Composition of a Selection of Plant-Based Ground Beef Alternative Products Available in the United States	Nutrients	Food Labels	Ground Beef	37	N/A	N/A	N/A	N/A

Curtain & Grafenauer	2019	Australia	Plant-based meat substitutes in the flexitarian age: An audit of products on supermarket shelves	Ingredients and Nutrients	Food labels	burgers, sausages, mince, chicken, seafood, and an additional ‘other’ category with products that fell outside of these categories (others: Meat-free products falling outside of other categories, including “Vegie Roast,” “Bacon-Style Rashers,” and “Polony)	137	More than two-thirds of all products (35%, n = 48) included legume ingredients, such as adzuki and black beans, chickpeas, and lentils, with products containing between 9%–65% legume ingredients.	Fat/Saturated Fat: Vegetable oil, canola oil, sunflower oil, sunflower kernels, rice bran oil, coconut oil, flax seed meal, cocoa butter, peanuts	protein: Soy protein, pea protein, soy beans, hydrolysed vegetable protein, mycoprotein, almonds	N/A

D'Alessandro et al.,	2022	Italy	Processed Plant-Based Foods for CKD Patients: Good Choice, but Be Aware	Ingredients and Nutrients	Food labels	Burgers, desserts and ice cream, cold cuts and sausages, cutles and nuggets, meatballs, ready meat, plant-based drinks, cheeses, yogurts, sauces	560	Soy is the most used ingredient, followed by rice and oats	Seed oil	N/A	The most used (>50%) are those belonging to the class of stabilizers/thickeners, followed by emulsifiers and, finally, by antioxidants. All the other classes were used in smaller percentages (<7%).
Bryngelsson et al.,	2022	Sweden	Nutritional assessment of plant-based meat analogues on the Swedish market	Ingredients and Nutrients	Food labels	Sausages, bites/fillets, burgers, mince, cold cuts, ball, schnitzel, nugget, bacon and others	142	Soy protein, pea protein, wheat, pulses	rapeseed and sunflower	Soy and pea	N/A

Tonheim et al.,	2022	Norway	Plant-based meat and dairy substitutes on the Norwegian market: comparing macronutrient content in substitutes with equivalent meat and dairy products	Nutrients	Foods labels	Plant-based meat and dairy substitutes	102 meat substitute products and 173 milk and dairy substitute products	N/A	N/A	N/A	N/A
Alessandrini et al.,	2021	UK	Nutritional Quality of Plant-Based Meat Products Available in the UK: A Cross-Sectional Survey	Nutrients	Foods labels	Plant-Based Meat Products (sausages, burgers, plain poultry, breaded poultry, mince, meatballs	207 Plant-based meat and 226 meat products	N/A	N/A	N/A	N/A

Cole et al.,	2021	USA	Examination of the nutritional composition of alternative beef burgers available in the United States	Nutrients	Food labels	Alternative beef burgers	Imitation (n = 28), veggie (n = 89), and conventional beef (n = 41) burgers	N/A	N/A	N/A	N/A

Table S2– Full data regarding the nutritional composition of the samples of the included studies

Original Study	Classification	Main Protein Source	Energy	Carbohydrates	Sugars	Protein	Dietary Fiber	Total Fat	Saturated Fat	Sodium	Iron	Zinc	Vitamin B12
Harnack et al.,	Minced	Various	153	12.7	0	12.6	1.9	6	1.3	265	10	0.7	0
Curtain & Grafenauer	Chicken Cutlets	Various	198	12.6	2.1	13.4	4.7	9.4	1.6	508	4.8	0	0
Curtain & Grafenauer	Burguers	Various	175	16.7	3.4	9.6	5.3	7.2	1.5	372	3.6	0	0
Curtain & Grafenauer	Sausages	Various	175	11.4	2.2	13.4	4.2	7.9	2.4	497	3.4	0	0
Curtain & Grafenauer	Others	Various	185	13	3.2	14.5	4.9	7.9	1.6	568	3.2	0	0
Curtain & Grafenauer	Minced	Various	137	7.9	1.9	13.7	5.9	5.4	2.1	401	2.8	0	0
Bryngelsson et al.,	Sausages	Soy and Pea Proteins	208	4.7	1.5	13	4.2	15.4	0	490	2.75	0	1.25

Bryngelsson et al.,	Burguers	Soy and Pea Proteins	208	8	1.1	14	3.8	13	0	420	2.75	0	0
Bryngelsson et al.,	Minced	Soy and Pea Proteins	171	9.7	1.05	15.55	5	9.7	0	320	2.63	0	0
Bryngelsson et al.,	Meat Balls	Soy and Pea Proteins	196	11.35	1.1	13.6	4.2	11.35	0	440	2.1	0	0.38
Bryngelsson et al.,	Schnitzel	Soy and Pea Proteins	196	11	1.2	17	5.5	11	0	440	2.1	0	0.38
Bryngelsson et al.,	Chicken Nuggets	Soy and Pea Proteins	217	10	1.1	16	5.3	10	0	420	2.1	0	0.38
Cole et al.,	Burguers	Various	189.57	10.36	0.79	18.21	4.11	9.43	2.36	398.39	0.078	0	0
Svarc et al.,	Cold Cuts	Mycroprotein	176	4.6	1.1	8	2.3	14.2	0	0	0	0	0
Svarc et al.,	Minced	Mycroprotein	109	7.7	0.2	14	6.5	2.5	0	0	0	0	0
Svarc et al.,	Minced	Pea Protein	230	9.5	1.9	16	5.6	14	0	0	0	0	0
Svarc et al.,	Sausages	Pea Protein	212	7.2	1.6	16	5.1	13	0	0	0	0	0
Svarc et al.,	Minced	Soy Protein	185	8.4	0.2	20	8.1	2.8	0	0	0	0	0
Svarc et al.,	Pieces	Soy Protein	136	8.4	0.2	20	8.1	2.8	0	0	0	0	0
Svarc et al.,	Sausages	Soy Protein	136	8.4	0.8	12	6.9	10	0	0	0	0	0
Svarc et al.,	Meat Balls	Soy Protein	171	9.3	1.2	14.8	7.7	8.4	0	0	0	0	0
Svarc et al.,	Cold Cuts	Soy and Pea Proteins	158	7.1	2.7	7.5	2.8	11	0	0	0	0	0
Svarc et al.,	Cold Cuts	Wheat Protein	231	17.5	5.9	3.1	5.5	4.6	0	0	0	0	0
Svarc et al.,	Pieces	Wheat Protein	198	5.3	0.7	28	2.3	7.2	0	0	0	0	0
Curtain & Grafenauer	Seafood	Various	160	15.1	3.3	8.9	3.6	7.6	1.4	447	0	0	0
D'Alessandro et al.,	Burguers	Various	178	11.9	1.8	12.3	4.3	8	1.3	400	0	0	0
D'Alessandro et al.,	Cold Cuts	Various	142	4.1	0.9	14.8	2.1	7.4	1.1	420	0	0	0
D'Alessandro et al.,	Cutlets	Various	196	15.7	0.9	10.1	3.5	9.4	1.2	420	0	0	0
D'Alessandro et al.,	Meat Balls	Various	211	14.6	1.8	11.4	4.5	10.9	1.4	420	0	0	0

Bryngelsson et al.,	Pieces	Soy and Pea Proteins	171	6.4	1.1	16.05	5	5.7	0	445	0	3	0
Bryngelsson et al.,	Cold Cuts	Soy and Pea Proteins	171	4.15	1.1	11	2.4	13.1	0	840	0	0	0
Romão et al.,	Burguers	Various	216	18.22	0	14.77	5.6	8.91	3.2	434	0	0	0
Romão et al.,	Minced	Various	192.81	12.91	0	14.25	5.77	10	3.01	572.96	0	0	0
Romão et al.,	Chicken Nuggets	Various	216.12	17.38	0	12.97	4.32	10.7	1.28	499.62	0	0	0
Romão et al.,	Chicken Cutlets	Various	201.92	10.07	0	18.25	6.08	9.29	5.04	372.38	0	0	0
Romão et al.,	Chicken Cutlets	Various	163.33	8.9	0	21.77	6.79	5.17	0.63	458.67	0	0	0
Romão et al.,	Seafood	Various	194.22	14.7	0	8.75	6.41	11	2.63	416.02	0	0	0
Romão et al.,	Seafood	Various	193.7	13.83	0	10.07	4.67	11.75	1.53	482.03	0	0	0
Romão et al.,	Sausages	Various	189.69	8.63	0	14.2	4.71	9.85	1.73	572.35	0	0	0
Romão et al.,	Cold Cuts	Various	251.97	14.57	0	19.64	4.71	9.85	1.73	572.35	0	0	0
Mariseva et al.,	Various	Various	228	7.5	1.9	22.4	0	11.3	2.2	1200	0	0	0
Boukid et al.,	Seafood	Various	208	3.6	0.5	14.9	0	10.4	1.8	320	0	0	0
Boukid et al.,	Seafood	Various	96	10.6	1.5	1	0	3.7	0.01	136	0	0	0
Boukid et al.,	Seafood	Various	202	11.88	0.99	1.09	0	4.26	0.64	196	0	0	0
Boukid et al.,	Seafood	Various	233	20	0.89	11.33	0	11.2	1.1	420	0	0	0
Boukid et al.,	Seafood	Various	243	25.35	1.4	10.63	0	11.43	1.1	556	0	0	0
Boukid et al.,	Seafood	Various	144	8.6	0.6	2.77	0	7.07	0.93	480	0	0	0
Boukid et al.,	Seafood	Various	13	1	0	1	0	0.5	0	1360	0	0	0
Boukid et al.,	Seafood	Various	209	15.4	0.8	13.4	0	8.9	1.1	240	0	0	0
Tonheim et al.,	Various	Various	201.5	8.4	1	13	3.6	10.2	1.1	600	0	0	0
Alessandrini et al.,	Sausages	Various	175.4	0	0	12.10	5.40	8.90	2.60	560	0	0	0
Alessandrini et al.,	Burguers	Various	203.1	0	0	11.10	4.60	10.30	1.70	440	0	0	0

Alessandrini et al.,	Chicken Cutlets	Various	161.5	0	0	18.70	5.60	5.70	0.80	520	0	0	0
Alessandrini et al.,	Chicken Nuggets	Various	233.9	0	0	13.20	5.10	11.00	1.30	480	0	0	0
Alessandrini et al.,	Minced	Various	170.3	0	0	20.80	5.60	5.70	1.70	280	0	0	0
Alessandrini et al.,	Meat Balls	Various	178.9	0	0	13.90	5.50	8.70	1.10	440	0	0	0
